# Clinical outcomes and lung mechanics characteristics between COVID-19 and non-COVID-19-associated acute respiratory distress syndrome: a propensity score analysis of two major randomized trials

**DOI:** 10.5935/0103-507X.20220040-en

**Published:** 2022

**Authors:** Bruno Martins Tomazini, Eduardo Leite Vieira Costa, Bruno Adler Maccagnan Pinheiro Besen, Fernando Godinho Zampieri, Carlos Roberto Ribeiro de Carvalho, Eliana Bernardete Caser, Vicente Cés de Souza-Dantas, Emerson Boschi, Renata Rego Lins Fumis, Meton Soares de Alencar Filho, Israel Silva Maia, Wilson de Oliveira Filho, Viviane Cordeiro Veiga, Alvaro Avezum, Renato Delascio Lopes, Flávia Ribeiro Machado, Otávio Berwanger, Regis Goulart Rosa, Alexandre Biasi Cavalcanti, Luciano César Pontes de Azevedo

**Affiliations:** 1 Hospital Sírio-Libanês - São Paulo (SP), Brazil.; 2 Medical Intensive Care Unit, Discipline of Clinical Emergencies, Hospital das Clínicas, Faculdade de Medicina, Universidade de São Paulo - São Paulo (SP), Brazil.; 3 Research Institute, HCor - Hospital do Coração - São Paulo (SP), Brazil.; 4 Respiratory Intensive Care Unit, Hospital das Clínicas, Faculdade de Medicina, Universidade de São Paulo - São Paulo (SP), Brazil.; 5 Unimed Vitória - Vitória (ES), Brazil; 6 Hospital Naval Marcílio Dias - Rio de Janeiro (RJ), Brazil.; 7 Hospital Geral Caxias do Sul - Caxias do Sul (RS), Brazil.; 8 Hospital Maternidade São Vicente de Paulo - Barbalha (CE), Brazil.; 9 Hospital Nereu Ramos – Florianópolis (SC), Brazil.; 10 Hospital e Pronto Socorro 28 de Agosto - Manaus (AM), Brazil.; 11 BP - A Beneficência Portuguesa de São Paulo - São Paulo (SP), Brazil.; 12 International Research Center, Hospital Alemão Oswaldo Cruz - São Paulo (SP), Brazil.; 13 Brazilian Clinical Research Institute - São Paulo (SP), Brazil.; 14 Discipline of Anesthesiology, Pain and Intensive Care, Escola Paulista de Medicina, Universidade Federal de São Paulo - São Paulo (SP), Brazil.; 15 Academic Research Organization, Hospital Israelita Albert Einstein - São Paulo (SP), Brazil.; 16 Hospital Moinhos de Vento - Porto Alegre (RS), Brazil.

**Keywords:** COVID-19, Coronavirus infections, Respiratory distress syndrome, Respiratory mechanics, Critical care, Critical care outcomes

## Abstract

**Objective:**

To compare the lung mechanics and outcomes between COVID-19-associated acute respiratory distress syndrome and non-COVID-19-associated acute respiratory distress syndrome.

**Methods:**

We combined data from two randomized trials in acute respiratory distress syndrome, one including only COVID-19 patients and the other including only patients without COVID-19, to determine whether COVID-19-associated acute respiratory distress syndrome is associated with higher 28-day mortality than non-COVID-19 acute respiratory distress syndrome and to examine the differences in lung mechanics between these two types of acute respiratory distress syndrome.

**Results:**

A total of 299 patients with COVID-19-associated acute respiratory distress syndrome and 1,010 patients with non-COVID-19-associated acute respiratory distress syndrome were included in the main analysis. The results showed that non-COVID-19 patients used higher positive end-expiratory pressure (12.5cmH2O; SD 3.2 *versus* 11.7cmH2O SD 2.8; p < 0.001), were ventilated with lower tidal volumes (5.8mL/kg; SD 1.0 *versus* 6.5mL/kg; SD 1.2; p < 0.001) and had lower static respiratory compliance adjusted for ideal body weight (0.5mL/cmH2O/kg; SD 0.3 *versus* 0.6mL/cmH2O/kg; SD 0.3; p = 0.01). There was no difference between groups in 28-day mortality (52.3% *versus* 58.9%; p = 0.52) or mechanical ventilation duration in the first 28 days among survivors (13 [IQR 5 - 22] *versus* 12 [IQR 6 - 26], p = 0.46).

**Conclusion:**

This analysis showed that patients with non-COVID-19-associated acute respiratory distress syndrome have different lung mechanics but similar outcomes to COVID-19-associated acute respiratory distress syndrome patients. After propensity score matching, there was no difference in lung mechanics or outcomes between groups.

## INTRODUCTION

Coronavirus disease 2019 (COVID-19)-associated acute respiratory distress syndrome (ARDS) has been perceived as a particular subtype of ARDS due to distinct pathophysiological features.^(1-3)^

Acute respiratory distress syndrome is a heterogeneous syndrome^(4,5)^ allowing for distinct subphenotype classifications based on clinical, physiological, and biological characteristics.^(6-8)^ This heterogeneity is acknowledged in the Berlin definition of ARDS,^(5)^ where patients are ultimately divided into three categories based on the partial pressure of oxygen to fraction of inspired oxygen (PaO_2_/FiO_2_) ratio, each category with distinct mortality risks. This possible oversimplification of ARDS phenotypes by oxygenation strata in the Berlin definition, together with clinical and pathophysiological particularities of severe COVID-19 disease, led to discussions of whether COVID-19-associated ARDS should be considered typical ARDS.^(3,9,10)^

Some reports suggest that patients with COVID-19-associated ARDS have higher respiratory system compliance for a given PaO_2_/FiO_2_ ratio than patients with non-COVID-19 ARDS,^(1,11,12)^ while others demonstrate similar lung mechanics in both scenarios.^(13,14)^ In addition, whether COVID-19-associated ARDS yields higher mortality than non-COVID-19 ARDS is still unclear.^(1,12,13,15)^ Additionally, there are no data specifically comparing patients with ARDS caused by pneumonia (pulmonary ARDS) with COVID-19 patients.

Therefore, in this analysis, we combined data from two randomized trials in ARDS,^(16,17)^ one including only COVID-19 patients and the other including only patients without COVID-19, to determine whether COVID-19-associated ARDS is associated with higher 28-day mortality than non-COVID-19 ARDS and to examine the differences in lung mechanics between these two types of ARDS.

## METHODS

### Study design and participants

We performed a secondary analysis of two randomized clinical trials involving patients with moderate or severe ARDS. The Alveolar Recruitment Trial (ART)^(17)^ was an international, multicenter, randomized pragmatic trial that included 1,010 patients diagnosed with ARDS according to the American-European Consensus Conference criteria from November 2011 through April 2017. Patients with early ARDS (< 72 hours) were included in the study if their PaO_2_/FiO_2_ ratio remained below 200mmHg at positive end-expiratory pressure (PEEP) ≥ 10cmH_2_O and FiO_2_ = 100% after at least 3 hours of ventilation, according to the low PEEP, low tidal volume ARDS Network ventilation protocol (ARMA protocol). Patients were excluded if any of the following criteria were met: age < 18 years; use of vasopressors in increasing doses in the last 2 hours; mean arterial pressure < 65mmHg; intracranial hypertension or acute coronary syndrome; pneumothorax, subcutaneous emphysema, pneumomediastinum or pneumatocele; and patients without therapeutic perspective and exclusive palliative care. Patients were randomized 1:1 to either protective mechanical ventilation according to the ARDSNet protocol^(18)^ or to a strategy that involved lung recruitment and PEEP titration according to the best compliance of the respiratory system.

The CoDEX^(16)^ study included 299 patients with moderate to severe ARDS caused by COVID-19 from April 2020 through June 2020. Patients were included within 48 hours of diagnosing moderate or severe ARDS according to the Berlin criteria. Exclusion criteria were age < 18 years, pregnancy or active lactation, allergy to dexamethasone, daily corticosteroid use 15 days prior to inclusion, indication for corticosteroid use other than ARDS, use of immunosuppressive drugs or other immunosuppression states, moribund patients and consent refusal. Patients were randomized 1:1 to receive standard of care or standard of care plus dexamethasone for 10 days. Protective ventilation protocols were encouraged but not protocolized. Of the 41 centers in the CoDEX trial, 21 (51.2%) also randomized patients in the ART trial.

### Variables

We extracted demographic, ventilatory, and gas exchange data after randomization in the ART trial and immediately after randomization in the CoDEX study (baseline data). We normalized static compliance to ideal body weight (IBW) to account for differences in lung sizes.^(19)^ We also calculated the ventilatory ratio, an index of ventilation efficiency, which is influenced by pulmonary dead space and carbon dioxide (CO_2_) production, where higher values (> 1) represent increased pulmonary dead space or increased CO_2_ production.^(20,21)^

### Outcomes

The primary outcome was 28-day mortality. Secondary outcomes included mechanical ventilation duration in the first 28 days and intensive care unit (ICU) length of stay (LOS) among survivors, ventilatory parameters (PEEP, FiO_2_ and tidal volume), PaO_2_/FiO_2_ ratio, and respiratory system mechanics.

### Statistical analysis

Continuous data are presented as the means and standard deviations (SDs) or medians and interquartile ranges (IQRs). Categorical data are presented as counts and percentages. Categorical variables were compared using Fisher’s exact test or Pearson’s χ^2^, while continuous variables were analyzed using the *t* test and Wilcoxon rank-sum test for normally and nonnormally distributed data, respectively. We used a multivariable logistic regression model to assess the association between COVID-19 status and 28-day mortality adjusted for age, sex, Simplified Acute Physiology Score (SAPS) 3, PaO_2_/FiO_2_ ratio, and ventilatory ratio.

Three analyses were performed. The main analysis included all patients from both trials (entire population analysis). The second included all patients from the CoDEX trial and all patients with pulmonary ARDS from the ART trial, defined as pneumonia being the primary insult leading to ARDS. The third analysis sought to reduce the effects of baseline factors and disease severity, which were expected since patients randomized in the ART trial had a stabilization period before enrollment, which led to the possible exclusion of patients who increased their PaO_2_/FiO_2_ ratio during the stabilization period. To account for possible imbalances, we created a propensity score to match patients with similar baseline characteristics from the two trials. For each patient, a propensity score indicating the likelihood of belonging to each trial (and therefore of being COVID-19 ARDS or non-COVID-19 ARDS) was calculated using a logistic regression model with the following variables: demographics (age and sex), an overall critical illness severity variable (SAPS 3), and the ARDS severity defining variable (PaO_2_/FiO_2_ ratio). We used this propensity score to match one to one patients from the two trials using the nearest-neighbor method using the optimal algorithm, without replacement, with the MatchIt package for R.^(22)^ Between-group comparisons after the propensity score method were carried out using the McNemar test for dichotomous variables and the paired *t* test or Wilcoxon rank-sum test for continuous variables as appropriate.^(23)^ In the case of partially paired data due to missing data, pooled *t* tests were used.^(24)^

Additionally, we performed a sensitivity analysis excluding all patients from the ART trial randomized to the lung recruitment group, which was associated with worse outcomes. All analyses were conducted using R, version 3.6.2. Two-sided p values ≤ 0.05 were considered significant, analyses were performed without imputation for missing data, and there was no adjustment for multiple comparisons.

## RESULTS

### Entire population analysis

All patients from both the ART^(17)^ (n = 1,010) and CoDEX^(16)^ (n = 299) trials were included in the entire population analysis. COVID-19 patients at baseline were older (mean age 61.4; SD 14.6 *versus* 50.9; SD 17.4; p < 0.001) and more severely ill (mean SAPS 3 70.3; SD 12.6 *versus* 63.2; SD 18.5; p < 0.001) than non-COVID-19 patients (Table 1).

**Table 1:** Baseline characteristics and outcomes of the entire population^*^†

	Non-COVID-19 (n = 1010)	COVID-19 (n = 299)	p value
Age (years)	50.9 (17.4)	61.4 (14.6)	< 0.001
Male (%)	631 (62.5)	187 (62.5)	> 0.99
SAPS 3	63.2 (18.5)	70.3 (12.6)	< 0.001
PaO_2_/FiO_2_ ratio (mmHg)	118.3 (42.7)	131.8 (45.9)	< 0.001
PaCO_2_ (mmHg)	57.8 (21.7)	47.5 (13.5)	< 0.001
Respiratory rate (ipm)	25.3 (6.4)	24.3 (5.4)	0.02
PEEP (cmH_2_O)	12.5 (3.2)	11.7 (2.8)	< 0.001
Plateau pressure (cmH_2_O)	25.9 (5.1)	23.9 (4.9)	< 0.001
Driving pressure† (cmH_2_O)	13.4 (4.5)	12.5 (3.4)	0.02
Tidal volume (mL/kg of IBW)	5.8 (1.0)	6.5 (1.2)	< 0.001
Static compliance‡ (mL/cmH_2_O/kg)	0.5 (0.3)	0.6 (0.3)	0.01
Ventilatory ratio	2.0 [1.5 - 2.7]	1.9 [1.5 - 2.5]	0.01
ARDS severity (%)			< 0.001
Moderate	599 (59.3)	216 (72.2)	
Severe	411 (40.7)	83 (27.8)	
MV duration (days)§	13 [8 - 20]	12 [6 - 26]	0.46
ICU LOS (days)¶	13 [5 - 22]	26 [22 - 28]	< 0.001
28-day mortality (%)	528 (52.3)	176 (58.9)	0.52

SAPS 3 - Simplified Acute Physiology Score 3; PaO_2_/FiO_2_ - partial pressure of oxygen to fraction of inspired oxygen; PaCO_2_ - partial pressure of carbon dioxide; ipm - incursions per minute; PEEP - positive end-expiratory pressure; IBW - ideal body weight; ARDS - acute respiratory distress syndrome; MV - mechanical ventilation; ICU - intensive care unit; LOS - length of stay.

* All data are from the day of randomization. The number of missing data on each variable for the ART and CoDEX trial is, respectively: PaCO_2_ 8 and 2; respiratory rate 0 and 24; PEEP 0 and 25; plateau pressure 1 and 125; driving pressure 1 and 139; tidal volume 0 and 63; static compliance 1 and 155; ventilatory ratio 8 and 64. † Driving pressure is the difference between plateau pressure and positive end-expiratory pressure. ‡ Weight-adjusted respiratory system static compliance is the ratio of tidal volume to driving pressure divided by ideal body weight. § Mechanical ventilation duration was evaluated only among survivors. ¶ Intensive care unit length of stay was evaluated only among survivors. Results expressed as mean (standard deviation) or median [interquartile range].

**Figure 1:**
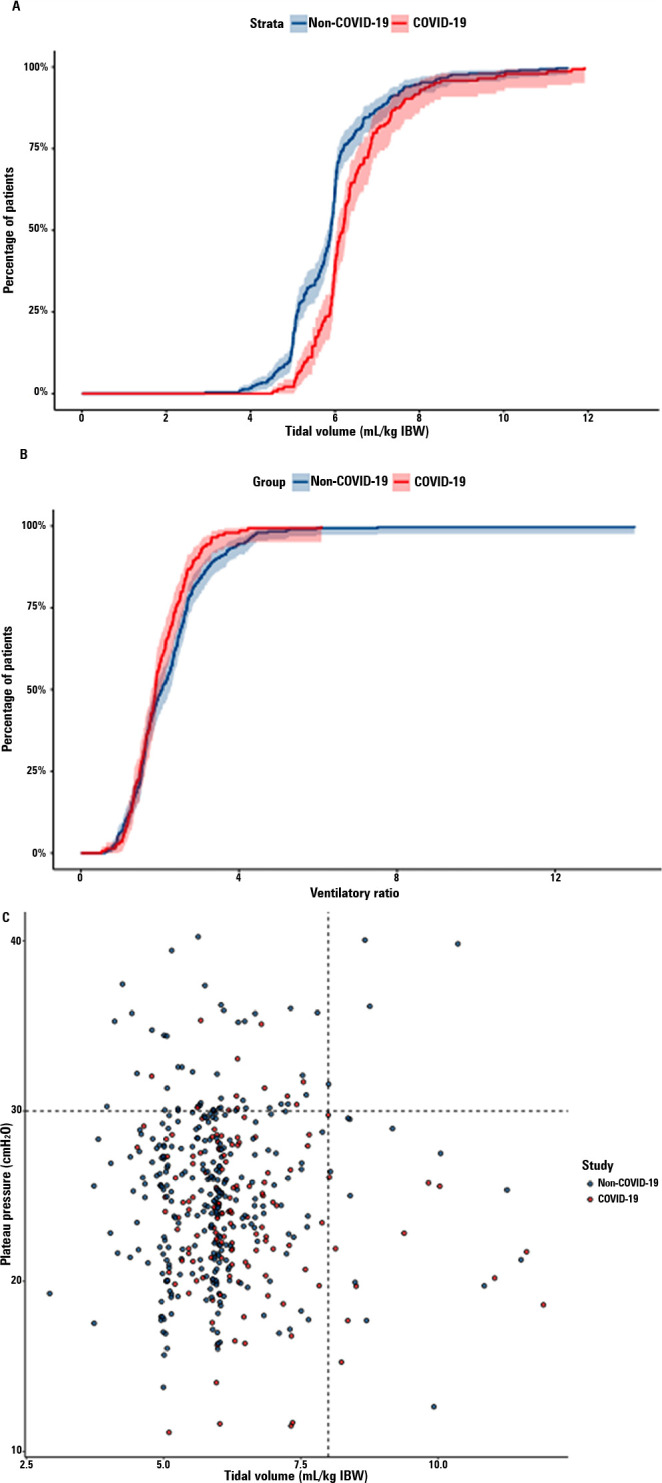
Ventilation parameters in the matched groups. (A) Cumulative distribution of tidal volume between matched groups. (B) Cumulative distribution of ventilatory ratio between matched groups. (C) Distribution of tidal volume vs. plateau pressure in the matched groups.

A higher proportion of non-COVID-19 patients had severe ARDS compared to COVID-19 patients. Non-COVID-19 patients had a lower PaO_2_/FiO_2_ ratio, used higher PEEP levels, were ventilated with lower tidal volumes, had higher driving pressures, had lower static respiratory compliance adjusted for IBW, and had higher PaCO_2_ (Table 1; Figure 1S - Supplementary material). Patients with non-COVID-19 ARDS had a higher ventilatory ratio than COVID-19 patients did.

There was no difference between groups in 28-day mortality (52.3% *versus* 58.9%; p = 0.52) or mechanical ventilation duration in the first 28 days among survivors (13 [IQR 5 - 22] *versus* 12 [IQR 6 - 26]; p = 0.46); however, 28-day mortality was higher in patients with COVID-19 and moderate ARDS (61.1% *versus* 51.3%; p = 0.016). COVID-19 status was not associated with an increased risk of 28-day mortality after adjusting for age, SAPS 3, ventilatory ratio, and PaO_2_/FiO_2_ ratio (Table 1S - Supplementary material).

### Pulmonary acute respiratory distress syndrome analysis

**Figure 2:**
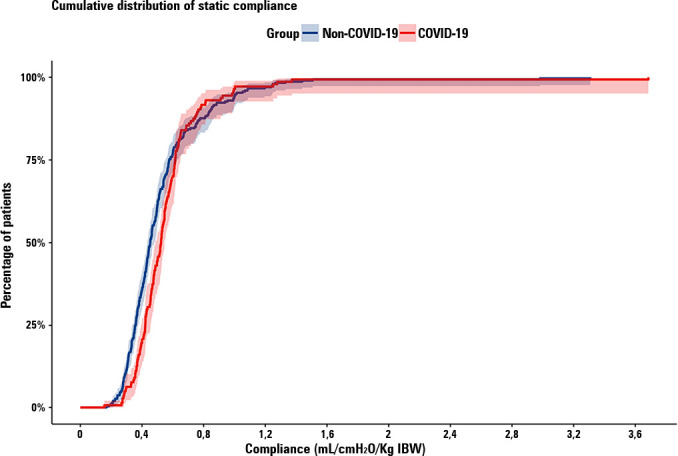
Cumulative distribution of static compliance adjusted for ideal body weight between matched groups.

A total of 556 patients in the ART trial had pneumonia as the primary insult leading to ARDS. At baseline, COVID-19 patients were older and had a higher SAPS 3 than non-COVID-19 patients with pulmonary ARDS. Non-COVID-19 patients with pulmonary ARDS had a lower PaO_2_/FiO_2_ ratio, used higher PEEP levels, were ventilated with lower tidal volumes, had higher driving pressures, had a higher ventilatory ratio, and had higher PaCO_2_. There was no difference between groups in static respiratory compliance adjusted for IBW, 28-day mortality, or mechanical ventilation duration in the first 28 days among survivors (Table 2S and Figure 2S - Supplementary material).

### Propensity matched analysis

The baseline and outcome data of the matched analysis are shown in table 2. There was no difference between the COVID-19 and non-COVID-19 groups regarding the matching variables age, SAPS 3, PaO_2_/FiO_2_ ratio, and gender. There was no difference in ARDS severity or PEEP levels between groups. The distribution of tidal volumes significantly differed between groups (Figure 1A), with non-COVID-19 patients being ventilated with lower tidal volumes (Figure 3S - Supplementary material).

(A) The cumulative distribution of tidal volume was lower in the non-COVID-19 group; however, the majority of patients in both groups received lung-protective ventilation with tidal volumes equal to or lower than 8mL/kg of ideal body weight. (B) The cumulative distribution of the ventilatory ratio was similar between groups. (C) Distribution of tidal volume vs. plateau pressure for each patient in which the data were available. Most patients fell within the limits of lung-protective ventilation (lower-left corner), defined as plateau pressure equal to or lower than 30cmH_2_O and tidal volume equal to or lower than 8mL/kg of ideal body weight.

**Table 2:** Baseline characteristics and outcomes of the matched population^*^

	Non-COVID-19 (n = 299)	COVID-19 (n = 299)	p value
Age (years)	61.3 (15.7)	61.4 (14.6)	0.94
Male (%)	190 (63.5)	187 (62.5)	0.87
SAPS 3	69.8 (18.5)	70.3 (12.6)	0.69
PaO_2_/FiO_2_ ratio (mmHg)	131.0 (42.9)	131.8 (45.9)	0.83
PaCO_2_ (mmHg)	57.8 (22.8)	47.5 (13.5)	< 0.001
Respiratory rate (ipm)	24.7 (6.4)	24.3 (5.4)	0.43
PEEP (cmH_2_O)	12.1 (3.0)	11.7 (2.8)	0.1
Plateau pressure (cmH_2_O)	25.3 (5.0)	23.9 (4.9)	0.003
Driving pressure† (cmH_2_O)	13.2 (4.6)	12.5 (3.4)	0.1
Tidal volume (mL/kg) of IBW	5.9 (1.2)	6.5 (1.2)	< 0.001
Static compliance‡ (mL/cmH_2_O/kg)	0.5 (0.3)	0.6 (0.3)	0.23
Ventilatory ratio	2.1 [1.5 - 2.7]	1.9 [1.5 - 2.5]	0.051
ARDS severity (%)			0.93
Moderate	214 (71.6)	216 (72.2)	
Severe	85 (28.4)	83 (27.8)	
MV duration (days)§	12 [7 - 20]	12 [6 - 26]	0.2
ICU LOS (days)¶	14 [5 - 21]	26 [22 - 28]	< 0.001
28-day mortality (%)	171 (57.2)	176 (58.9)	0.74

SAPS 3 - Simplified Acute Physiology Score 3; PaO_2_/FiO_2_ - partial pressure of oxygen to fraction of inspired oxygen; PaCO_2_ - partial pressure of carbon dioxide; ipm - incursions per minute; PEEP - positive end-expiratory pressure; IBW - ideal body weight; ARDS - acute respiratory distress syndrome; MV - mechanical ventilation; ICU - intensive care unit; LOS - length of stay.

* All data are from the day of randomization. † Driving pressure is the difference between plateau pressure and positive end-expiratory pressure. ‡ Weight-adjusted respiratory system static compliance is the ratio of tidal volume to driving pressure divided by ideal body weight. § Mechanical ventilation duration was evaluated only among survivors. ¶ Intensive care unit length of stay was evaluated only among survivors. Results expressed as mean (standard deviation) or median [interquartile range].

In the matched population, there was no significant difference between the non-COVID-19 and COVID-19 groups in driving pressure (Figure 4S - Supplementary material), static respiratory compliance adjusted for IBW (Figure 2), or ventilatory ratio (Figure 1B). Most patients in both groups received lung-protective ventilation, defined as plateau pressure equal to or less than 30cmH_2_O and tidal volume equal to or less than 8mL/kg IBW (Figure 1C).

There was no statistically significant difference in the cumulative frequency distribution of static respiratory compliance adjusted for ideal body weight between groups (p = 0.23). IBW - ideal body weight.

There was no difference in 28-day mortality or mechanical ventilation duration in the first 28 days among survivors between the COVID-19 and non-COVID-19 groups. COVID-19 patients had a longer ICU LOS in the first 28 days among survivors (26 [IQR 22 - 28] days *versus* 14 [IQR 5 - 21] days; p < 0.001).

### Sensitivity analysis

The sensitivity analysis excluding all patients randomized to the lung recruitment strategy (Table 3S - Supplementary material) showed no difference between groups in 28-day mortality (53.0% *versus* 58.9%; p = 0.12) or mechanical ventilation duration in the first 28 days among survivors (median 13 [IQR 8 - 20] days *versus* 12 [IQR 6 - 26] days; p = 0.55).

## DISCUSSION

In this secondary analysis of two randomized clinical trials in ARDS patients, we observed similar 28-day mortality between COVID-19-associated ARDS and non-COVID-19 ARDS in the entire population analysis, the pulmonary ARDS analysis, and the propensity score-matched analysis. Furthermore, there was no difference in mechanical ventilation duration among survivors, which reinforces the similarities between COVID-19 and other causes of ARDS.

We observed comparable 28-day mortality between groups in the entire population, pulmonary ARDS and matched analyses. The high mortality rate in all analyses might be explained by the severity of illness, as shown by high SAPS 3, which might also explain the mortality differences between our study and others.^(1,25)^ Additionally, there was no difference between groups in mechanical ventilation duration in the first 28 days among survivors, which goes against the subjective impression that intensivists might have, which might be a form of recall bias, that COVID-19 patients have longer mechanical ventilation duration. These findings suggest that COVID-19 causes ARDS with similar patient-centered outcomes compared to typical ARDS.

We also observed that COVID-19 ARDS and typical ARDS behaved similarly regarding respiratory system mechanics and gas exchange. In the entire population analysis and the pulmonary ARDS analysis, non-COVID-19 patients had lower static compliance. However, due to imbalances between the two populations before matching, such as a lower PaO_2_/FiO_2_ ratio in non-COVID-19 patients and a higher age and severity in the COVID-19 patients, nonadjusted comparisons of these two populations could be misleading. After propensity score matching for age, SAPS 3, gender, and PaO_2_/FiO_2_ ratio, there was no difference in static respiratory compliance or driving pressure between groups.

This finding challenges the hypothesis that most patients with COVID-19 have near-normal lung compliance.^(3)^ One explanation for this misperception might be that COVID-19 patients with near-normal lung compliance and a low PaO_2_/FiO_2_ ratio, as described in other studies,^(1)^ might represent patients who were prematurely intubated,^(26)^ leading to discussions of whether these patients should have been intubated. In line with this reasoning is the published secondary analysis of lung-protective ventilation trials that demonstrated a higher benefit of low tidal volume ventilation among patients with lower respiratory system compliance. In contrast, higher tidal volumes could be tolerated in patients with higher compliance to allow spontaneous breathing while mechanically ventilated or even possibly allow safe ventilation under noninvasive respiratory support.^(27)^ Additionally, we adjusted static lung compliance to ideal body weight to account for differences in lung sizes,^(19)^ in contrast to other studies. Finally, the propensity score matching, which took into account ARDS and illness severity, is more robust than other analyses performed, allowing us to conclude that there is no difference in static lung compliance between COVID-19 and non-COVID-19 ARDS.

Most patients from both studies received lung-protective ventilation, and COVID-19 patients were ventilated with slightly higher tidal volumes and lower PEEP. In our study, the ventilatory ratio was high and similar between matched groups. This finding supports the notion that increased dead space is frequent in ARDS, irrespective of whether the underlying etiology is COVID-19. In the ART trial, the PEEP level was set based on the protocol (for both groups) and the tidal volume was strictly controlled; the differences in PEEP and tidal volume between the CoDEX and ART trials might be due to different protocols and processes of care.

Our study has limitations. First, although it used data from randomized clinical trials in ARDS, it was a retrospective study. Second, the CoDEX trial was performed in a pandemic context with overwhelmed health care systems, which might have led to worse results in the COVID-19 group. Conversely, the large volume of COVID-19-related ARDS cases cared for in a short time would arguably result in increased experience and standardization of care for those patients. Third, prone positioning was not part of the standard care when the ART trial was conducted, although it increased from 10% at baseline in the ART trial to only 22% in the CODEX trial. Fourth, mortality data were available only until 28 days after randomization, which might underestimate the real mortality rate in these populations. Fifth, our data only refer to variables in the peri-randomization period, not allowing for assessment of the dynamics of the disease process. Sixth, while the ART trial evaluated a mechanical ventilation intervention and had a stabilization period before enrollment, the CoDEX trial did not. This might have led to baseline differences in severity between groups since, after the stabilization period, less severe cases in the ART trial might have been excluded. Nevertheless, propensity score matching would mitigate this issue. Finally, since the study population of the two trials has statistically significant differences between demographic characteristics and outcomes, unmeasured confounders cannot be excluded despite the careful adjustment. Additionally, the lack of statistical power should be considered in the interpretation of the study findings.

## CONCLUSION

Our findings support the inclusion of COVID-19 among the etiologies of “typical” acute respiratory distress syndrome. The similarities of COVID-19 acute respiratory distress syndrome to acute respiratory distress syndrome from other causes far outweigh the differences, suggesting that standard of care ventilatory management can be applied.
