# Supplementary material for: Clinical outcomes and lung mechanics characteristics between COVID-19 and non-COVID-19-associated acute respiratory distress syndrome: a propensity score analysis of two major randomized trials
Source: Rev Bras Ter Intensiva. 2022 Jul-Sep;34(3):335–41. doi: 10.5935/0103-507X.20220040-en (PMC9749099; doi:10.5935/0103-507X.20220040-en)
Supplement: Supplementary file 1 [file rbti-34-03-0335-suppl01.pdf]

Clinical outcomes and lung mechanics characteristics between COVID-19 and non-COVID-19 associated acute respiratory distress syndrome: a propensity score analysis of two major randomized trials

Desfechos clínicos e características da mecânica pulmonar entre a síndrome do desconforto respiratório agudo associada à COVID-19 e a não associada à COVID-19: uma análise de escore de propensão de dois importantes ensaios randomizados

Bruno Martins Tomazini[1], Eduardo Leite Vieira Costa[1], Bruno Adler Maccagnan Pinheiro Besen[2], Fernando Godinho Zampieri[3], Carlos Roberto Ribeiro de Carvalho[4], Eliana Bernardete Caser[5], Vicente Cés de Souza-Dantas[6], Emerson Boschi[7], Renata Rego Lins Fumis[1], Meton Soares de Alencar Filho[8], Israel Silva Maia[9], Wilson de Oliveira Filho[10], Viviane Cordeiro Veiga[11], Alvaro Avezum[12], Renato Delascio Lopes[13], Flávia Ribeiro Machado[14], Otávio Berwanger[15], Regis Goulart Rosa[16], Alexandre Biasi Cavalcanti[3], Luciano César Pontes de Azevedo[1]

Table 1S - Multivariable logistic regression model evaluating predictors of 28-day mortality in the entire population

	Odds ratio (95%CI)	p value
Study (CoDEX)	0.9 (0.60 - 1.42)	0.73
Age (years)	1.02 (1.01 - 1.03)	< 0.001
SAPS 3	1.02 (1.01 - 1.03)	< 0.001
PaO_2/FiO_2 ratio	0.99 (0.99 - 1.0)	0.18
Ventilatory ratio	1.04 (0.93 - 1.17)	0.41
Male	0.92 (0.72 - 1.17)	0.50
Intervention	0.91 (0.70 - 1.18)	0.49
Interaction Study (CoDEX)*Intervention (Steroids)	0.97 (0.53 - 1.76)	0.93

SAPS 3 - Simplified Acute Physiology Score 3; PaO_2/FIO_2 - partial pressure of oxygen to fraction of inspired oxygen.

Table 2S - Baseline characteristics and outcomes of patients with pulmonary acute respiratory distress syndrome in COVID-19 and non-COVID-19 population[a,b]

	Non-COVID-19 (n = 556)	COVID-19 (n = 299)	p value
Age (years)	50.5 (17.7)	61.4 (14.6)	< 0.001
Male (%)	355 (63.8)	187 (62.5)	0.76
SAPS 3	63.1 (18.5)	70.3 (12.6)	< 0.001
PaO_2/FIO_2 ratio (mmHg)	121.0 (42.7)	131.8 (45.9)	0.001
$PaCO_2$ (mmHg)	58.5 (22.5)	47.5 (13.5)	< 0.001
Respiratory rate (ipm)	25.3 (6.2)	24.3 (5.4)	0.028
PEEP (cmH_2O)	12.6 (3.1)	11.7 (2.8)	< 0.001
Plateau pressure (cmH_2O)	26.0 (5.2)	23.9 (4.9)	< 0.001
Driving pressure [c] (cmH_2O)	13.4 (4.7)	12.5 (3.4)	0.03
Tidal Volume (mL/kg) of IBW	5.8 (1.1)	6.5 (1.2)	< 0.001
Static compliance [d] ($mL/cmH_2O/kg$)	0.5 (0.3)	0.6 (0.3)	0.05
Ventilatory ratio	2.1 [1.5-2.7]	1.9 [1.5-2.5]	0.01
ARDS severity (%)			0.004
Moderate	346 (62.2)	216 (72.2)	
Severe	210 (37.8)	83 (27.8)	
MV duration, days [e] median [IQR])	13 [8 - 21]	12 [6 - 26]	0.48
ICU LOS (days) [f] median [IQR]	13 [5 - 21]	26 [22 - 28]	< 0.001
28-day mortality (%)	301 (54.1)	176 (58.9)	0.21

SAPS 3 - Simplified Acute Physiology Score 3; PaO_2/FIO_2 - partial pressure of oxygen to fraction of inspired oxygen; $PaCO_2$ - partial pressure of carbon dioxide; IPM - incursion per minute; PEEP - positive end-expiratory pressure; IBW - ideal body weight; ARDS - acute respiratory distress syndrome; MV - mechanical ventilation; ICU - intensive care unit; LOS - length of stay.
[a] Continuous variables are presented as mean (standard deviation) unless otherwise indicated. [b] All data are from the day of randomization. [c] Driving pressure is the difference between plateau pressure and positive end expiratory pressure. [d] Weight adjusted respiratory system static compliance is the ratio of tidal volume to driving pressure divided by ideal body weight. [e] Mechanical ventilation duration was evaluated only among survivors. [f] Intensive care unit length of stay was evaluated only among survivors.

Table 3S - Baseline characteristics and outcomes of patients included in the sensitivity analysis[a,b]

	Non-COVID-19 (n = 509)	COVID-19 (n = 299)	p value
Age (years)	51.0 (17.4)	61.4 (14.6)	< 0.001
Male (%)	318 (62.5)	187 (62.5)	1.0
SAPS 3	62.8 (18.7)	70.3 (12.6)	< 0.001
PaO_2/FIO_2 ratio (mmHg)	117.8 (42.7)	131.8 (45.9)	< 0.001
$PaCO_2$ (mmHg)	56.5 (19.5)	47.5 (13.5)	< 0.001
Respiratory rate (ipm)	25.4 (6.3)	24.3 (5.4)	0.01
PEEP (cmH_2O)	12.5 (3.1)	11.7 (2.8)	< 0.001
Plateau pressure (cmH_2O)	25.7 (5.0)	23.9 (4.9)	< 0.001
Driving pressure [c] (cmH_2O)	13.2 (4.4)	12.5 (3.4)	0.09
Tidal volume (mL/kg) of IBW	5.8 (1.0)	6.5 (1.2)	< 0.001
Static compliance [d] ($mL/cmH_2O/kg$)	0.5 (0.3)	0.6 (0.3)	0.07
Ventilatory ratio	2.1 [1.6 - 2.7]	1.9 [1.5 - 2.5]	0.05
ARDS severity (%)			< 0.001
Moderate	301 (59.1)	216 (72.2)	
Severe	208 (40.9)	83 (27.8)	
MV duration, days [e] - median [IQR]	13 [8 - 20]	12 [6 - 26]	0.55
ICU LOS - days [f], median [IQR]	13 [6 - 22]	26 [22 - 28]	< 0.001
28-day mortality (%)	270 (53.0)	176 (58.9)	0.12

SAPS 3 - Simplified Acute Physiology Score 3; PaO_2/FIO_2 - partial pressure of oxygen to fraction of inspired oxygen; $PaCO_2$ - partial pressure of carbon dioxide; IPM - incursion per minute; PEEP - positive end-expiratory pressure; IBW - ideal body weight; ARDS - acute respiratory distress syndrome; MV - mechanical ventilation; ICU - intensive care unit; LOS - length of stay.
[a] Continuous variables are presented as mean (standard deviation) unless otherwise indicated. [b] All data are from the day of randomization. [c] Driving pressure is the difference between plateau pressure and positive end expiratory pressure. [d] Weight adjusted respiratory system static compliance is the ratio of tidal volume to driving pressure divided by ideal body weight. [e] Mechanical ventilation duration was evaluated only among survivors. [f] Intensive care unit length of stay was evaluated only among survivors.

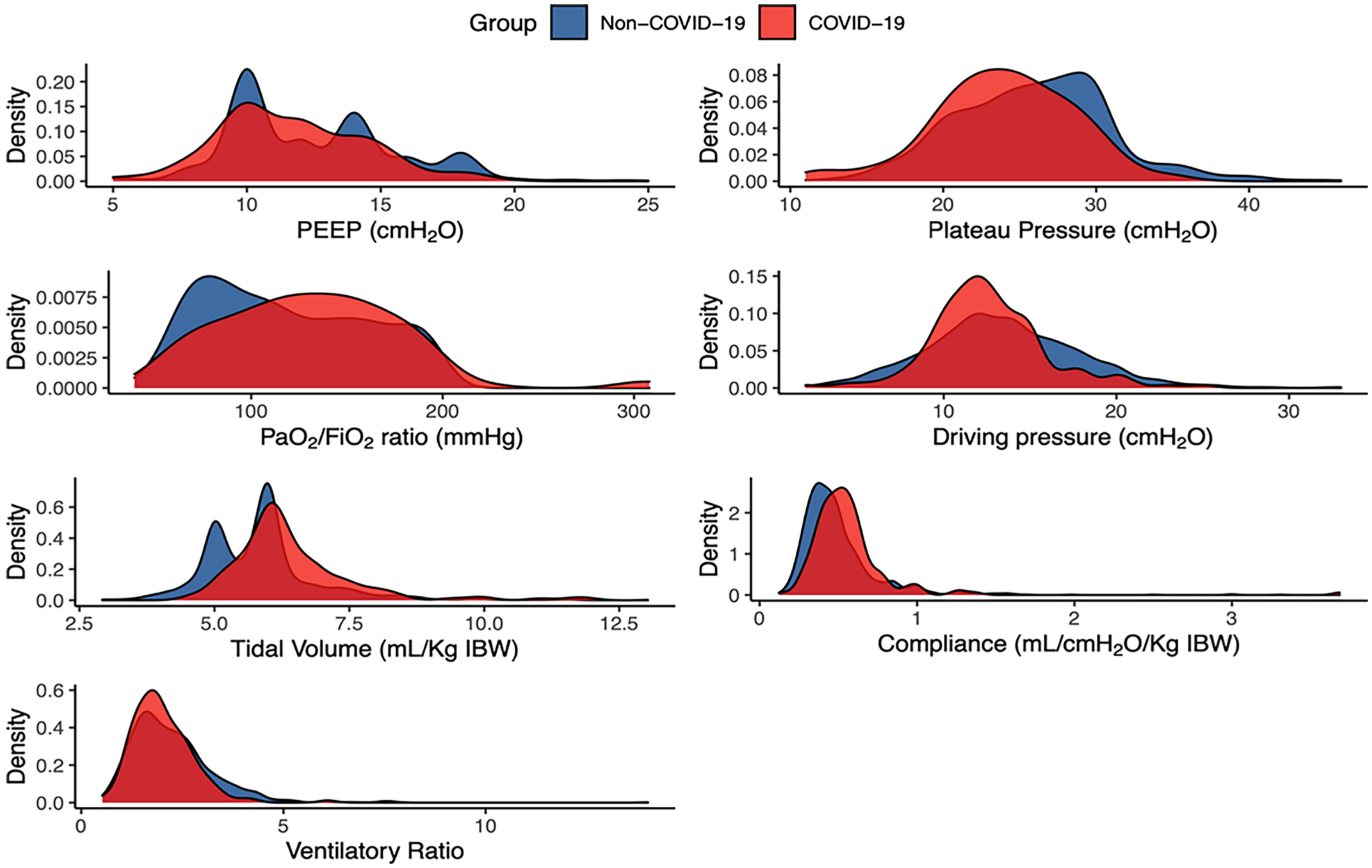

Figure 1S - Mechanical ventilation and respiratory parameters in COVID-19 and non-COVID-19 groups.
PEEP - positive end-expiratory pressure; PaO2/FIO2 - partial pressure of oxygen to fraction of inspired oxygen.

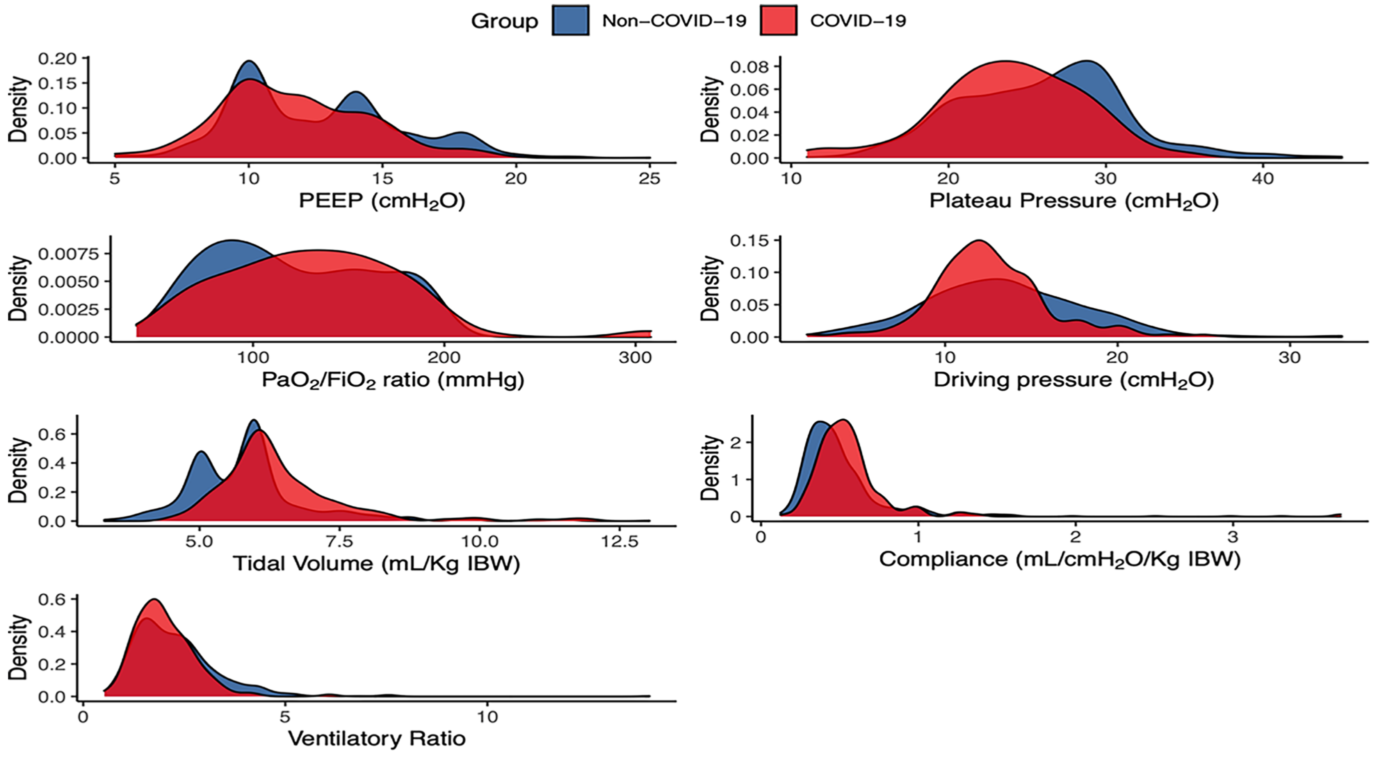

Figure 2S - Mechanical ventilation and respiratory parameters of patients with pulmonary acute respiratory distress syndrome in COVID-19 and non-COVID-19 population.

PEEP - positive end-expiratory pressure; PaO_2/FIO_2 - partial pressure of oxygen to fraction of inspired oxygen.

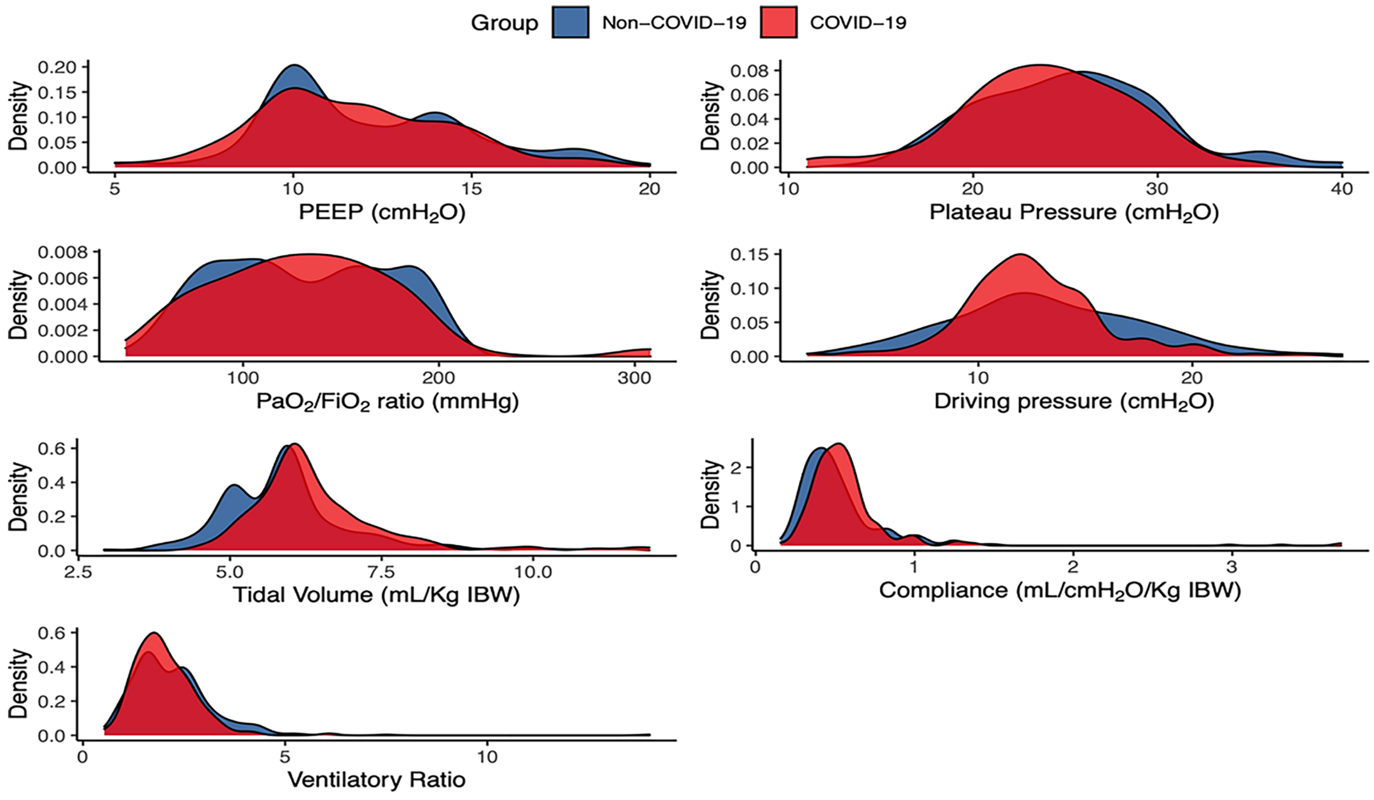

Figure 3S - Mechanical ventilation and respiratory parameters in matched population of COVID-19 and non-COVID-19 groups.

PEEP - positive end-expiratory pressure; PaO_2/FIO_2 - partial pressure of oxygen to fraction of inspired oxygen.

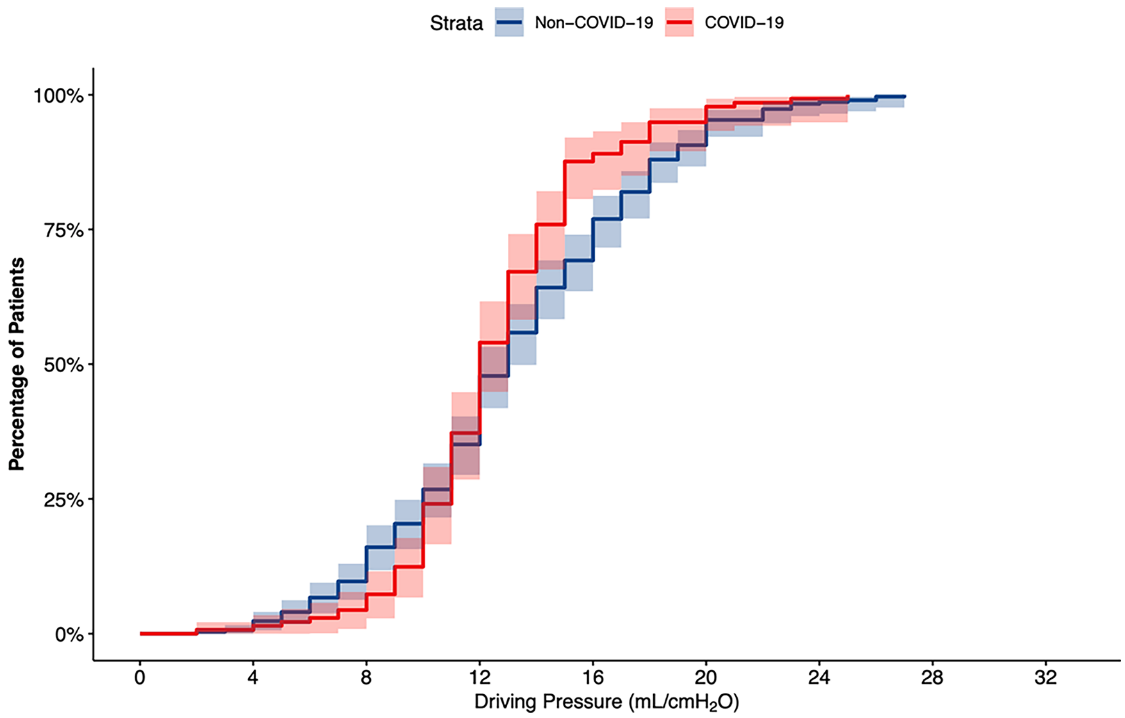

Figure 4Ss - Cumulative distribution of driving pressure between matched groups.

Cumulative distribution of driving pressure was similar between groups, with the majority of patients in both groups with driving pressure equal or lower than 16cmH2O.

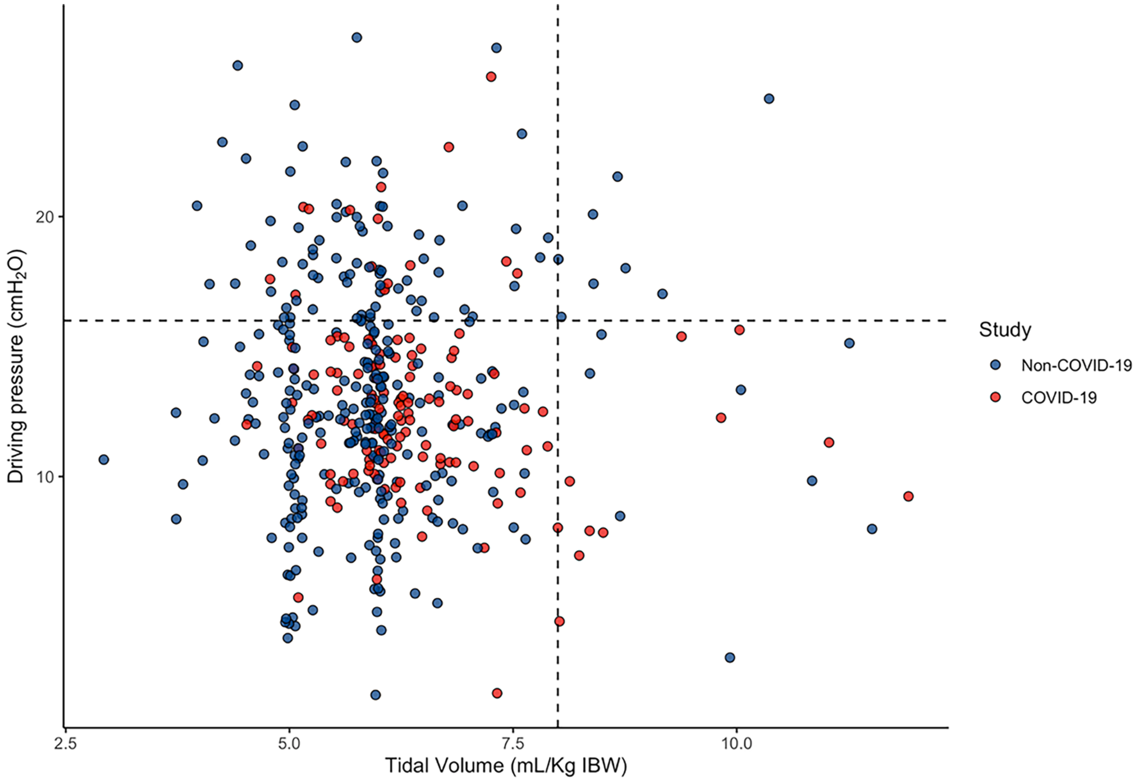

Figure 5S - Distribution of tidal volume *versus* driving pressure in the matched groups.

The graphic represents the distribution of tidal volume vs. driving pressure for each patient in which the data was available. The majority of patients fell within the limits of lung protective ventilation, defined as driving pressure equal or lower than 16cmH2O and tidal volume equal or lower than 8mL/kg of ideal body weight.

IBW - ideal body weight.

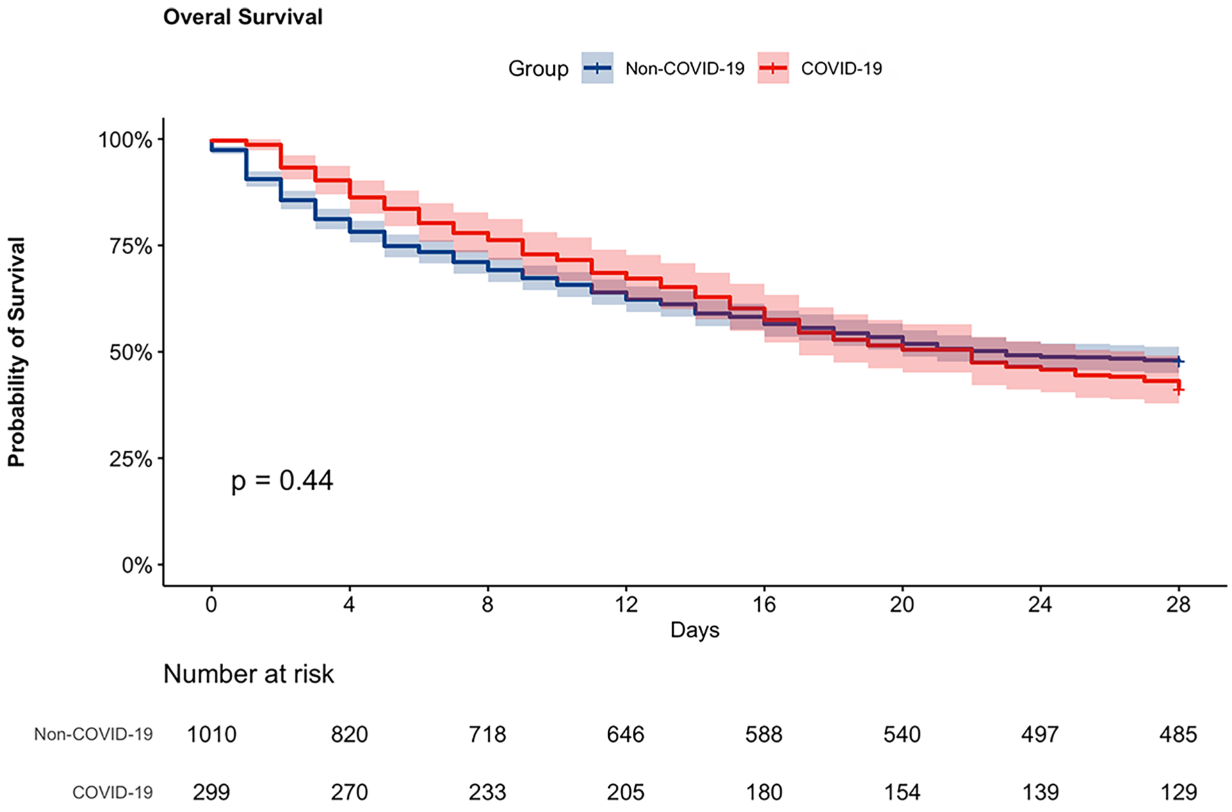

Figure 6S - Kaplan-Meier curves for 28-day survival in the entire population analysis.

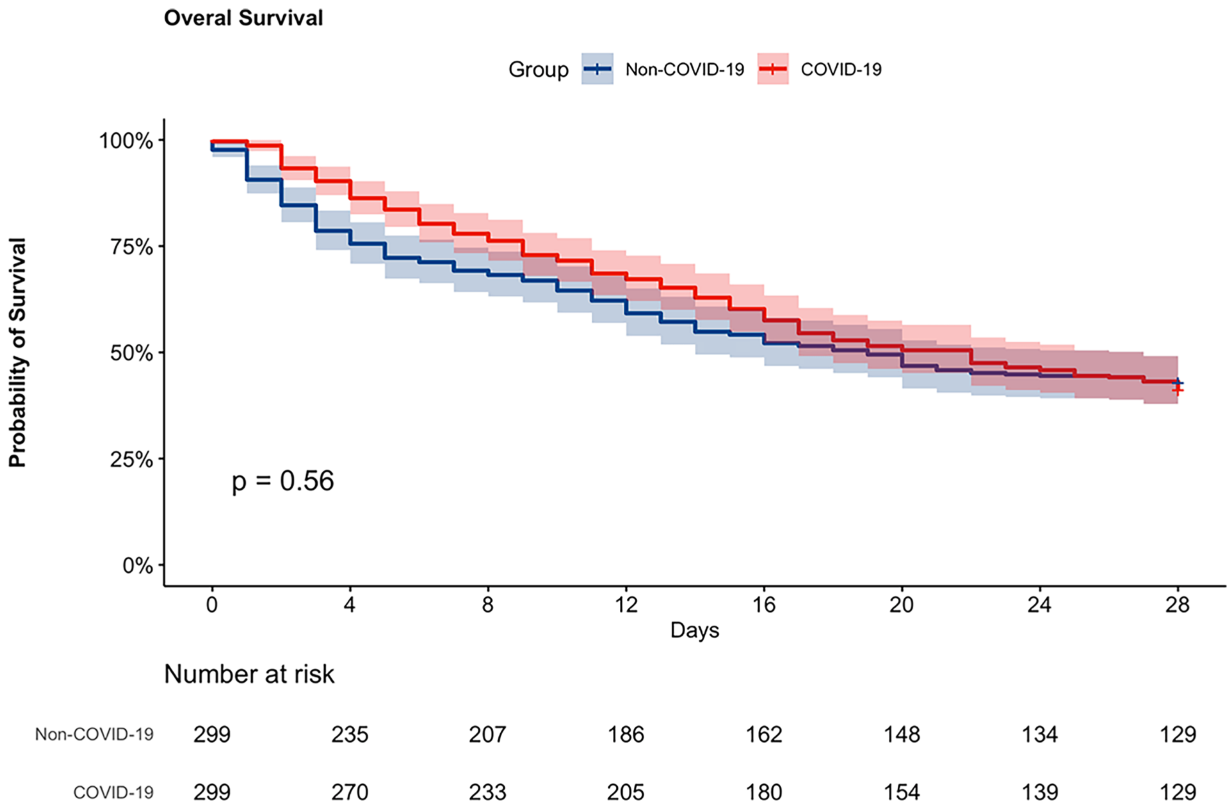

Figure 7S - Kaplan-Meier curves for 28-day survival in the propensity matched analysis

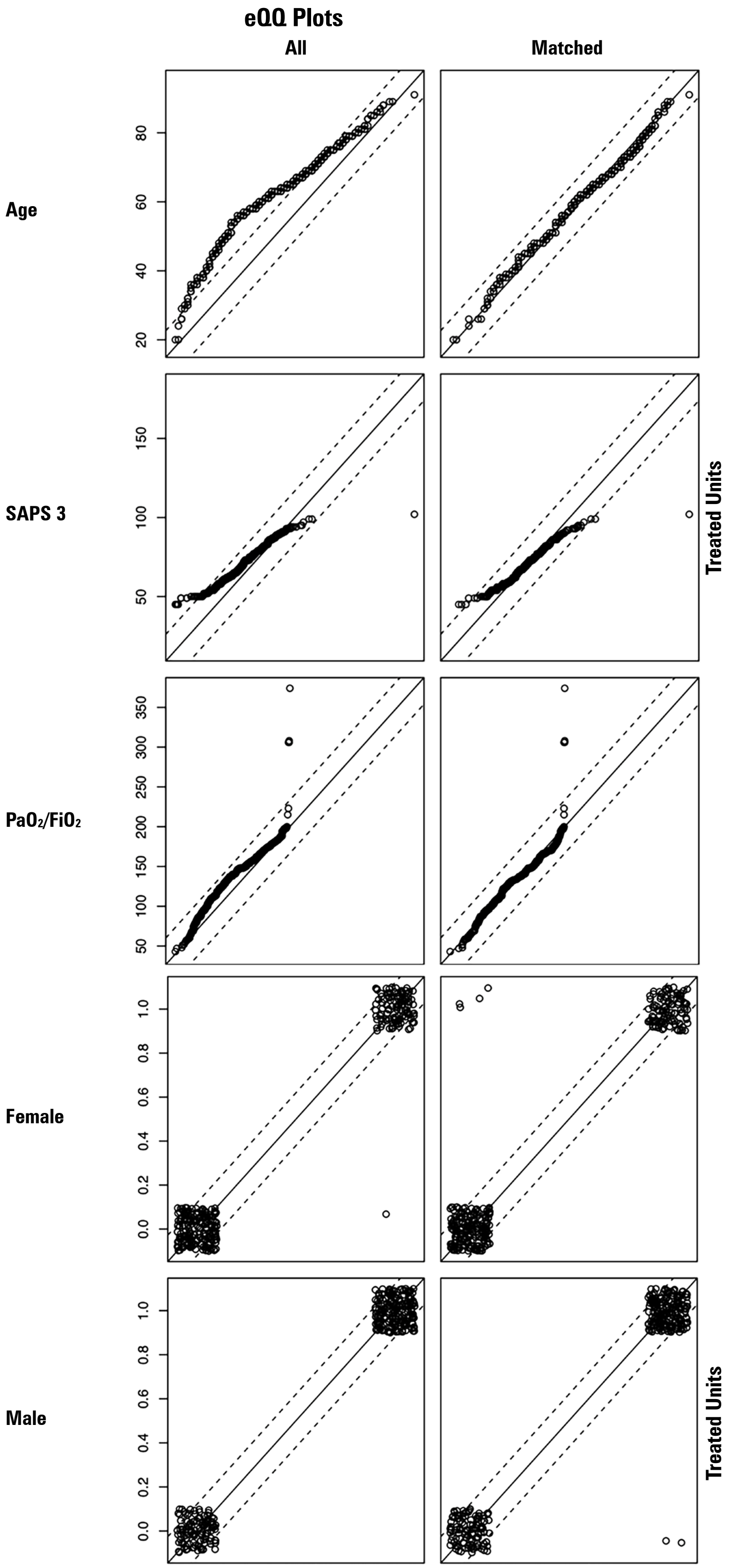

Figure 8S - Propensity score diagnostic plots (QQ Plot).

SAPS 3 - Simplified Acute Physiology Score 3; PaO_2/FiO_2 - partial pressure of oxygen to fraction of inspired oxygen.

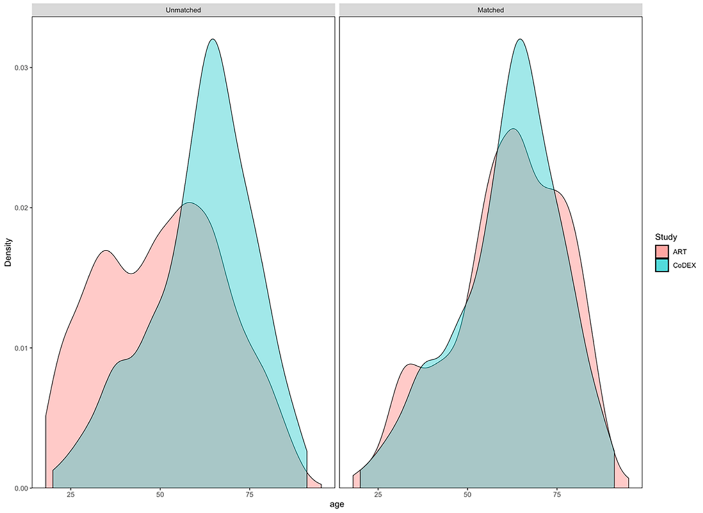

A

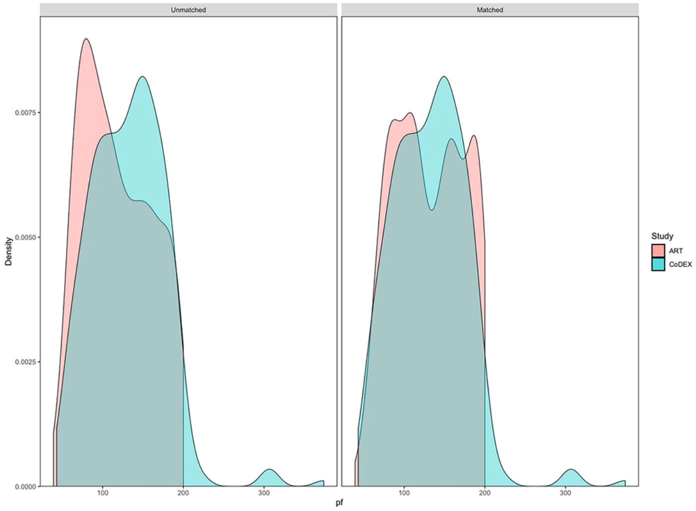

B

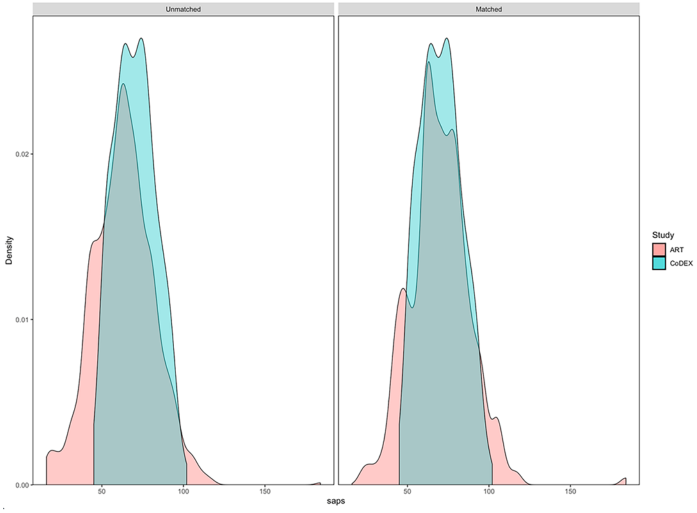

C

Figure 9S - Propensity score diagnostic plots (Distributional balance of variables). (A) Distributional balance for age. (B) Distributional balance for PaO_2/FiO_2 ratio. (C) Distributional balance for SAPS 3 score.

PaO_2/FIO_2 - partial pressure of oxygen to fraction of inspired oxygen; SAPS 3 - Simplified Acute Physiology Score 3; ART - Alveolar Recruitment Trial; CODEX - COVID-19 dexamethasone.
